# The morphology of VO_2_/TiO_2_(001): terraces, facets, and cracks

**DOI:** 10.1038/s41598-020-78584-9

**Published:** 2020-12-23

**Authors:** Jon-Olaf Krisponeit, Simon Fischer, Sven Esser, Vasily Moshnyaga, Thomas Schmidt, Louis F. J. Piper, Jan Ingo Flege, Jens Falta

**Affiliations:** 1 Institute of Solid State Physics, University of Bremen, 28359 Bremen, Germany; 2 MAPEX Center for Materials and Processes, University of Bremen, 28359 Bremen, Germany; 3 Experimentalphysik VI, Universität Augsburg, 86159 Augsburg, Germany; 4 I. Physikalisches Institut, Georg-August-Universität Göttingen, 37077 Göttingen, Germany; 5 WMG, Warwick University, Warwick, Coventry CV4 7AL UK; 6 Applied Physics and Semiconductor Spectroscopy, Brandenburg University of Technology Cottbus-Senftenberg, 03046 Cottbus, Germany

**Keywords:** Surfaces, interfaces and thin films, Structure of solids and liquids

## Abstract

Vanadium dioxide (VO_2_) features a pronounced, thermally-driven metal-to-insulator transition at 340 K. Employing epitaxial stress on rutile $\text{TiO}_{2}(001)$ substrates, the transition can be tuned to occur close to room temperature. Striving for applications in oxide-electronic devices, the lateral homogeneity of such samples must be considered as an important prerequisite for efforts towards miniaturization. Moreover, the preparation of smooth surfaces is crucial for vertically stacked devices and, hence, the design of functional interfaces. Here, the surface morphology of $\text{VO}_2/\text{TiO}_2(001)$ films was analyzed by low-energy electron microscopy and diffraction as well as scanning probe microscopy. The formation of large terraces could be achieved under temperature-induced annealing, but also the occurrence of facets was observed and characterized. Further, we report on quasi-periodic arrangements of crack defects which evolve due to thermal stress under cooling. While these might impair some applicational endeavours, they may also present crystallographically well-oriented nano-templates of bulk-like properties for advanced approaches.

## Introduction

Featuring a metal-insulator transition^1,2^, which ist observed at $T_{\text{MI}} = 340\,\text{K}$ for bulk samples, vanadium dioxide ($\text{VO}_{2}$) is considered a promising functional material for oxide electronic applications^3–7^ and functional coatings^8^. Thin films offer tunability of the electronic properties of transition metal oxides by substrate-induced biaxial strain^9^. Indeed, when exposed to coherent epitaxial strain, the transition temperature of $\text{VO}_{2}$ thin films can be altered over a remarkably wide range^10,11^. $\text{VO}_2(110)$ films on rutile $\text{TiO}_{2}(110)$ substrates have shown elevated transition temperatures close to $T_{\text{MI}} = 400\,\text{K}$^10,12^, due to a tensile in-plane strain of $3.6\%$ along the rutile *c*-axis, and pseudomorphic growth under strain as large as $8.7\%$ could recently be achieved on $\text{RuO}_{2}(110)$ substrates^13^. By contrast, a tensile stress in the rutile *ab*-plane as small as $0.8\%$, as experienced on $\text{TiO}_{2}(001)$ substrates, has been shown to inversely cause a reduction of $T_{\text{MI}}$ into the vicinity of room temperature, facilitating technological use.

Within the scope of this letter, two essential prerequisites for the successful fabrication of novel oxide electronic devices based on $\text{VO}_{2}(001)$ are investigated: (1) The quality and stability of the $\text{VO}_{2}(001)$ surface is studied. While previous studies mainly addressed the interface quality between substrate and $\text{VO}_{2}$ films, for instance investigating ion interdiffusion phenomena^14^ and the formation of misfit dislocations, little is known about the surface of $\text{VO}_{2}(001)$. Yet, its thorough understanding and control would be crucial for preparing vertically stacked devices where atomically smooth interfaces are required to ensure well-defined electrical top contacts and to enable fabrication of functional oxide interfaces^15^. (2) It is well known that $\text{VO}_{2}$ single crystals are prone to cracking when undergoing the transition^16,17^, as are sufficiently thick $\text{VO}_{2}(001)$ films under tensile epitaxial stress^18^. Although such crack defects must be considered as a severe obstacle for technological use, in this letter we discuss also their applicational potential for self-organized patterning.

Two different types of epitaxial $\text{VO}_{2}/\text{TiO}_{2}(001)$ films were investigated: First, ultrathin films ($\approx$ 5–10 nm) have been deposited in an ultrahigh vacuum (UHV) system by reactive molecular beam epitaxy (rMBE). These samples have been morphologically characterized in situ by conventional low-energy electron diffraction (LEED) and scanning tunneling microscopy (STM). While such in situ studies on the rMBE samples provide the highest cleanliness, the small achievable deposition rates hinder the growth of thick films. Therefore, a much thicker film ($\approx 85 \,\text{nm}$) was prepared by metal-organic aerosol deposition^19^ (MAD) ex situ and studied by low-energy electron microscopy (LEEM) and $\upmu$LEED. For this sample, the larger film thickness suggests that imperfections at the film-to-substrate interface, like Ti interdiffusion^14^, do not affect the surface morphology. Contrasting the significantly reduced transition known for $\text{VO}_{2}$ thin films on $\text{TiO}_2(001)$, transport data for the thick film show a transition temperature $T_{MI}\approx 340\,\text{K}$, i.e. close to the bulk value, and a rather broad hysteresis loop (see Supplementary Fig. S1). This indicates a partial and inhomogeneous stress release in the sample, due to, for instance, misfit dislocations. Hence, at room temperature, the rMBE is expected to be in the monoclinic state, whereas the MAD sample must be considered rutile. A comparison of these two sample types additionally allows for excluding effects specific to the chosen growth method.

Because the in situ grown rMBE films showed only diffuse LEED spots at the beginning and STM revealed a grainy morphology (see Supplementary Fig. S2), a short post-annealing at $300\,^{\circ }\text{C}$ was applied to coalesce the grains and obtain much sharper diffraction patterns. Similarly, the same annealing recipe was used to clean the ex situ MAD films from contaminations and to compensate for an overoxidation suffered from the contact with ambient atmosphere. An extensive description of the experimental techniques and procedures is given at the end of this article.

**Figure 1:**
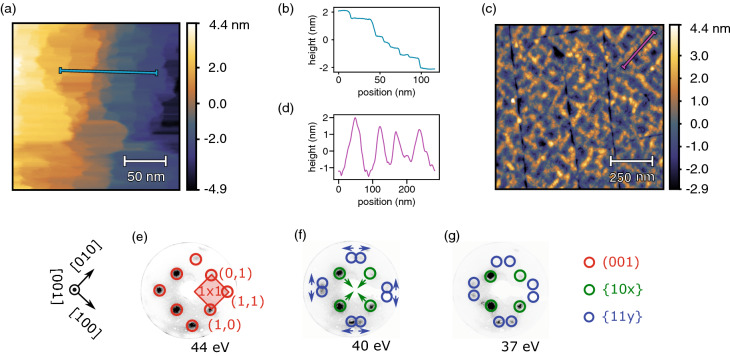
Surface morphology of $\text{VO}_{2}$ films grown by rMBE and MAD. (**a**) STM images reveal large $\text{(001)}$ terraces on the rMBE films after thermal annealing. (**b**) The height profile, corresponding to the path highlighted in blue in the image, shows integer atomic steps between the terraces. (**c**) AFM images of the MAD film exhibit a ripple-like texture. (**d**) The corresponding height profile depicts a slightly inclined (stepped) (001) surface. (**e**–**g**) LEED patterns of the rMBE films at different energies show a rutile $(1\times 1)$ reconstruction (**e**) but also feature $\{10x\}$ and $\{11y\}$ facet reflections (**f**,**g**).

## Results and discussion

### Surface morphology

After growth and annealing, both sample types exhibit similar diffraction patterns in LEED. Most importantly, a $(1\times 1)$ reconstruction, attributed to the desired flat $\text{VO}_2(001)$ termination^20^, can be observed regardless of their respective rutile and monoclinic bulk phases at room temperature, as well as for elevated temperatures. Note that for simplicity we here adhere to the rutile crystallographic basis which allows a uniform nomenclature for substrate and film. Exhibiting identical spot positions as for the $\text{TiO}_2(001)$ substrates, the observed $(1\times 1)$ indicates pseudomorphic growth of the $\text{VO}_2(001)$ films.

#### Ultrathin in situ films

For the rMBE samples, STM images accordingly confirmed the existence of atomically flat terraces, separated by integer steps of the rutile *c* lattice constant and with typical lateral dimensions of several tens of nm (see Fig. 1a,b). For some areas, even much larger terraces (up to $\approx 400\,\text{nm}$) were found. A corresponding series of LEED images is given in Fig. 1e–g: In agreement with the STM results, a clear $(1\times 1)$ reconstruction originating from these terraces could be observed for an electron energy of $44\,\text{eV}$ (Fig. 1e), approximately matching Bragg’s law for the third order of reflection at $\approx 42\,\text{eV}$ and an out-of-plane lattice constant $c\approx 0.285\,\text{nm}$. In addition to this $(1\times 1)$ reconstruction, however, also evidence for the formation of surface facets was revealed by LEED spots shifting their position with varying electron energy. Since these facet beams appear rather weak in LEED, and no such facet planes could be directly observed in the STM images, their occurrence is limited to a small fraction of the surface. The MBE samples showed a grainy appearance in STM before annealing (see Supplementary Fig. S2). We therefore attribute the facet reflections to small regions on the original grains which have been already aligned with the energetically favorable facet orientations and which hence have a higher stability against annealing. As indicated in Fig. 1f,g, the facet spots visible at reduced electron energies can be attributed to $\{10x\}$ and $\{11y\}$ types of facet planes.

#### Thick ex situ samples

Comparable diffraction patterns have been recorded by $\upmu$LEED also for the MAD sample. These will be characterized in detail later on, enabling an identification of the dominant facet types. Much sharper diffraction spots have been observed for this sample type due to the significantly higher growth temperature. Yet, this sample may also have suffered from the thermal instability of the unfavorable (001) surface, giving rise to more pronounced facet beams. Such behavior is well known for the closely related case of the rutile $\text{TiO}_{2}(001)$ surface, which undergoes a faceting transition for temperatures above $400\,^{\circ }\text{C}$^21^. Indeed, the MAD grown sample revealed a slightly rougher surface in AFM. Apart from crack-like features discussed later in this article, a texture of ripple-like objects is visible (Fig. 1c). The corresponding height profile (Fig. 1d), however, shows these ripples to have inclinations of only approximately $5^\circ$ with respect to the flat (001) plane. Hence, a stepped (001) termination must still be considered as the dominant surface motif. The orientation of the ripples along $<\!110\!>$-like directions might be understood as the onset of faceting, with step edges aligning to energetically favorable orientations.

To summarize, both sample types exhibit very similar surface morphologies with a coexistence of (001)-terminated regions and several types of facets. Subtle differences in roughness and the ripple-like feature found only for the MAD film are attributed to the considerably different growth temperatures. While the MAD sample shows an advanced faceting due to the thermal instability of the (001) termination, a similar behavior may be expected for MBE samples when grown at higher temperatures as well. The above findings, i.e. the occurrence of a $(1\times 1)$ reconstruction as well as indications for temperature-induced faceting, agree well with the results reported by Goering et al. for the $\{001\}$ surfaces of $\text{VO}_{2}$ single crystals^20^ as well as theoretical studies predicting the (001) surface to be rather unfavorable^22^. It should also be mentioned that a related formation of $\{110\}$-like facets was found on the $\text{VO}_{2}(100)$ surface by transmission electron microscopy^23^. Nonetheless, the fact that even an elevated growth temperature of $560\,^{\circ }\text{C}$ did not induce a severely faceted surface, unlike it is documented for $\text{TiO}_{2}$, is a promising finding for applicational efforts.

**Figure 2:**
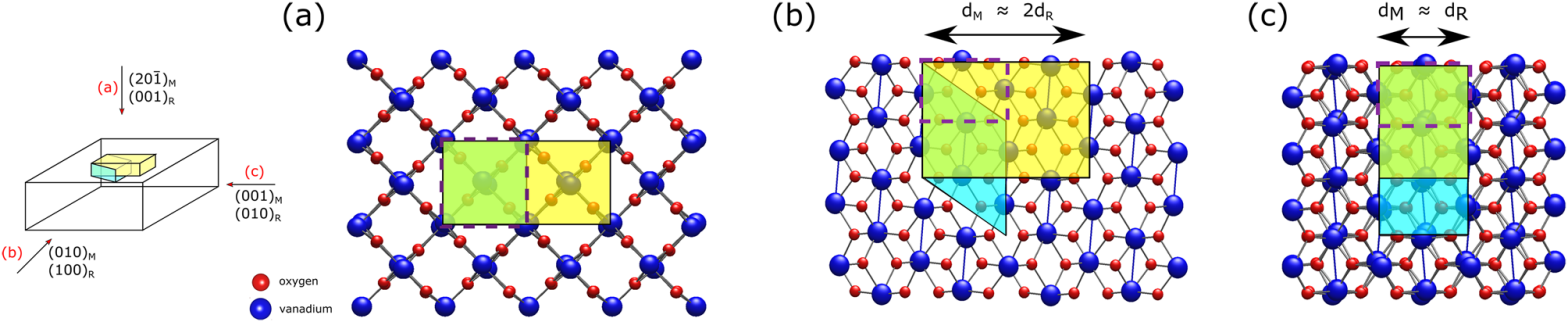
Surface periodicity of the monoclinic $(20\bar{1})$ surface. An enlarged $(1\times 2)$ periodicity is expected with respect to the rutile (001) surface. Crystallographic (shaded blue) und surface unit cells (yellow) are highlighted in (**a**) the top view onto the monoclinic $(20\bar{1})_\text{M}$/rutile $(001)_\text{R}$ surface, cross sections of (**b**) the $(010)_\text{M}/(100)_\text{R}$ and (**c**) the $(001)_\text{M}/(010)_\text{R}$ plane illustrate the monoclinic cell dimensions. The dashed rectangles outline a rutile cell. The sketch on the left indicates the respective lines of sight. The crystal structures were visualized by the open source software Vesta 3.4.7 (http://jp-minerals.org/vesta/en/)^24^.

### The ideal (001) surface

Before focussing on an identification of the relevant facet types, the desired (001) surface itself should be discussed in more detail. Interestingly, the $(1\times 1)$ reconstruction was observed below the transition temperature for both sample types while, assuming the monoclinic low-temperature phase $\text{VO}_{2}$ (M1), one could expect a $(1\times 2)$ periodicity (or an apparent $(2\times 2)$ in LEED due to averaging of rotational domains). As illustrated in Fig. 2, the flat (001) surface, ${(20\bar{1})}$ in monoclinic notation, actually possesses a $(1\times 2)$ larger surface unit mesh with respect to the rutile *ab*-plane, and hence LEED reflections could also be expected at the half-integer positions with respect to the $\text{TiO}_2(001)$ pattern. Yet, both the metallic and the insulating phase show the same $(1\times 1)$ reconstruction instead. Consequently, LEED patterns cannot be considered as a reliable method to track the sample state through the MIT. For an explanation of this behavior, several scenarios may be taken into account: (1) Originating from a rather subtle structural rearrangement, the super-structure reflections may appear too weak to be recorded by LEED. (2) Particularly when grown on $\text{TiO}_2(001)$ substrates, also a structural deviation from the monoclinic M1 structure may be considered for the low-temperature phase: Qiu et al. have recently obtained evidence for the existence of a “tetragonal-like” structural modification of the low-temperature phase by x-ray diffraction on comparable samples and proposed a structural model which indeed comprises a $(1\times 1)$ periodicity with respect to the rutile *ab*-plane^25^. (3) Finally, recent investigations by Wahila et al. provide experimental and computational evidence that the very surface may actually not even participate in the structural transition at all and prefer to remain rutile regardless^23^. As this observation appears valid even for $\text{VO}_{2}$ surfaces of distinct orientations it hence may be considered as a generic feature.

### Determination of facet orientations

When discussing the faceting tendency, a thorough comparison with the closely related and extensively studied case of rutile $\text{TiO}_{2}$ appears conclusive^26^. For both materials, though nonpolar, the ${\{001\}}$ surfaces must be considered as energetically unfavorable due to a large density of broken bonds. Density functional theory confirmed the $\text{VO}_{2}(001)$ surface to have the second-highest surface energy among the low-indexed surfaces orientations^22^. Consequently, a tendency to form facets has been reported for both systems. In early studies on $\text{TiO}_{2}$, the dominant $\{011\}$ and $\{114\}$ facets have been identified^27,28^, several additionally involved facet species, i.e. $\{023\}$, $\{045\}$, and $\{111\}$, were reported later^21^. As stated above, a related faceting tendency of the $\text{VO}_2(001)$ termination was briefly documented^20^ but the facet orientations have not been determined so far.

**Figure 3:**
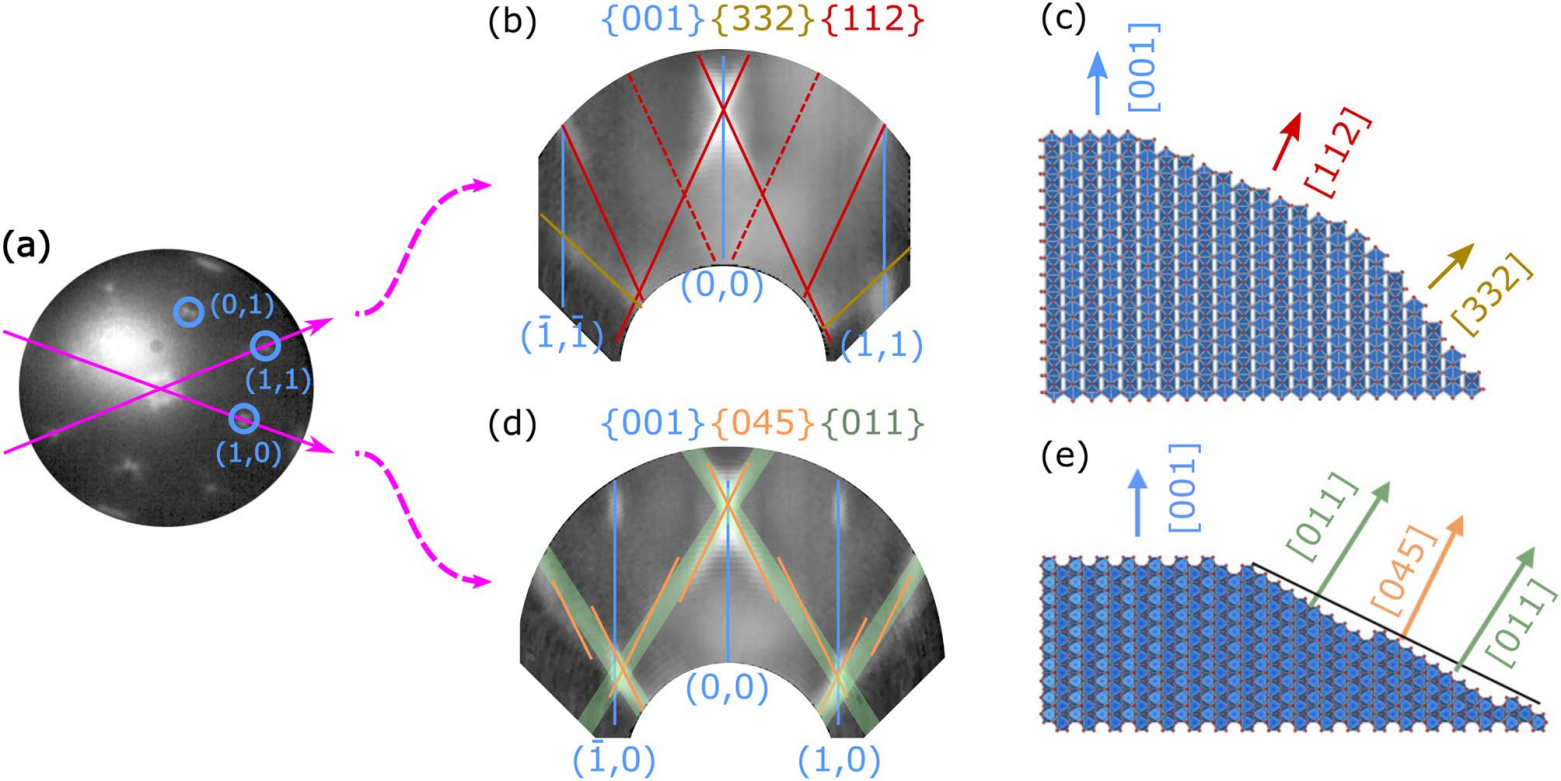
Identification of facet types on the $85\,\text{nm}$ thick $\text{VO}_{2}$ film. (**a**) Exemplary $\upmu$LEED pattern recorded at $23\,\text{eV}$, the highlighted paths illustrate the orientations of the generated reciprocal space maps. The full LEED stack is provided as Supplementary video 1. (**b**,**d**) Facet directions have been identified in the RSMs and corresponding structural models are given in (**c**,**e**). The structure models were created by the open source software Vesta 3.4.7 (http://jp-minerals.org/vesta/en/)^24^.

Therefore, in order to identify the relevant facet types, reciprocal space maps (RSMs) of the MAD sample have been created from a series of $\upmu$LEED images in the energy range from 6 to $25\,\text{eV}$ and with the electron beam being restricted to a diameter of ${5\,\upmu \text{m}}$. An exemplary $\upmu$LEED pattern is given is given in Fig. 3a. RSMs have been generated for the $(1\bar{1}0)$ and (010) reciprocal planes, shown in Fig. 3b,d, respectively. Note that these represent all $\{110\}$ and $\{100\}$ plane types by crystallographic symmetry.

#### Reciprocal space map of a (110)-like plane

In the first RSM (Fig. 3b), vertical rods are observed at the $(\bar{1},\bar{1})$, (0, 0) and (1, 1) LEED positions, indicating the existence of a flat (001)-oriented surface component. In addition, facet reflections have been observed under angles of approximately $25^\circ \pm 1^\circ$ and $43^\circ \pm 2^\circ$. As illustrated in the morphological model shown in Fig. 3c, the angles agree with the occurrence of facets oriented in the $\{112\}$ (expected at $24^\circ$ for bulk $\text{VO}_{2}$) and presumably $\{332\}$ directions. The $\{332\}$ facets appear much weaker in the RSM, due to their large inclination, i.e. the smaller illumination density in LEED of the projection of the facets to the (001) direction. As they also show a lack of symmetry, due to spatial aberrations of the electron optics significantly affecting the outer regions of the recorded $\upmu$LEED patterns, this angle is more difficult to determine and the identification as $\{332\}$ is considered tentative. For the $\{112\}$ facets, dominant reflections occur with a spacing of $\approx 0.145\mathring{\text{A}}^{-1}$, or $\approx 0.69\,\text{nm}$ in real space. Yet, much weaker, an increased intensity is also observed at the half spacing. These spacings would correspond well with $(1\times 1)$ or $(1\times 2)$ terminated facets, respectively, as a periodicity of ${0.705\,\text{nm}}$ would be expected for $(1\times 1)$.

#### Reciprocal space map of a (100)-like plane

The second RSM (Fig. 3d) shows vertical rods at the $(\bar{1},0)$, (0, 0) and (1, 0) LEED positions, again corresponding to the flat (001) surface component. However, also rather broad streaks of higher intensity are observed under an angle of approximately $33^\circ \pm 1^\circ$ (indicated in green). These can be regarded as a series of narrower sub-streaks at an angle of $26^\circ \pm 1^\circ$ (marked in orange). The latter can be assigned to facet planes in the (045) direction with their narrow spacing reflecting their large unit mesh. The enhanced intensity along (011) in broad streaks can be understood considering this high-indexed plane as as a regularly stepped (011) plane instead. The measured angles are again in good agreement with the expected inclinations of $32.1^\circ$ for $\{011\}$ facets and $26.6^\circ$ for $\{045\}$ facets. The spacing of $\approx 0.35\mathring{\text{A}}^{-1}$ between the $\{011\}$ reflections suggests a spatial periodicity of $\approx 0.28\,\text{nm}$ in the facet. Being only about half the surface mesh dimension along the corresponding direction ($\approx 0.54\,\text{nm}$) this may appear contradictory at first sight. However, a glide mirror plane perpendicular to the rutile (011) surface must be taken into account which leads to the observed extinction of every second rod in the RSM. Again, a morphological model is given to illustrate the facet orientations (Fig. 3e).

Hence, alike its well-studied relative, rutile $\text{TiO}_{2}$, the $\text{VO}_2(001)$ surface is prone to an irreversible formation of facets. Yet, as noted before, it features a higher stability: While $\text{TiO}_2(001)$ becomes unstable already above $\approx 400\,^\circ \text{C}$^21^. the stepped (001) surface can be obtained on $\text{VO}_{2}$ samples grown at temperature of at least up to $560\,^\circ \text{C}$. Beyond this enhanced robustness, which may prove very valuable for applicational purposes, also differences in the preferred facet types exist: As on rutile titania, ${\{045\}}$ planes, i.e. stepped ${\{011\}}$ facets, have been identified as relevant terminations. However, while rather flat ${\{114\}}$ facets are common on $\text{TiO}_{2}$, we report indications for the occurrence of much steeper ${\{112\}}$ and, tentatively, ${\{332\}}$ terminations on $\text{VO}_2(001)$.

**Figure 4:**
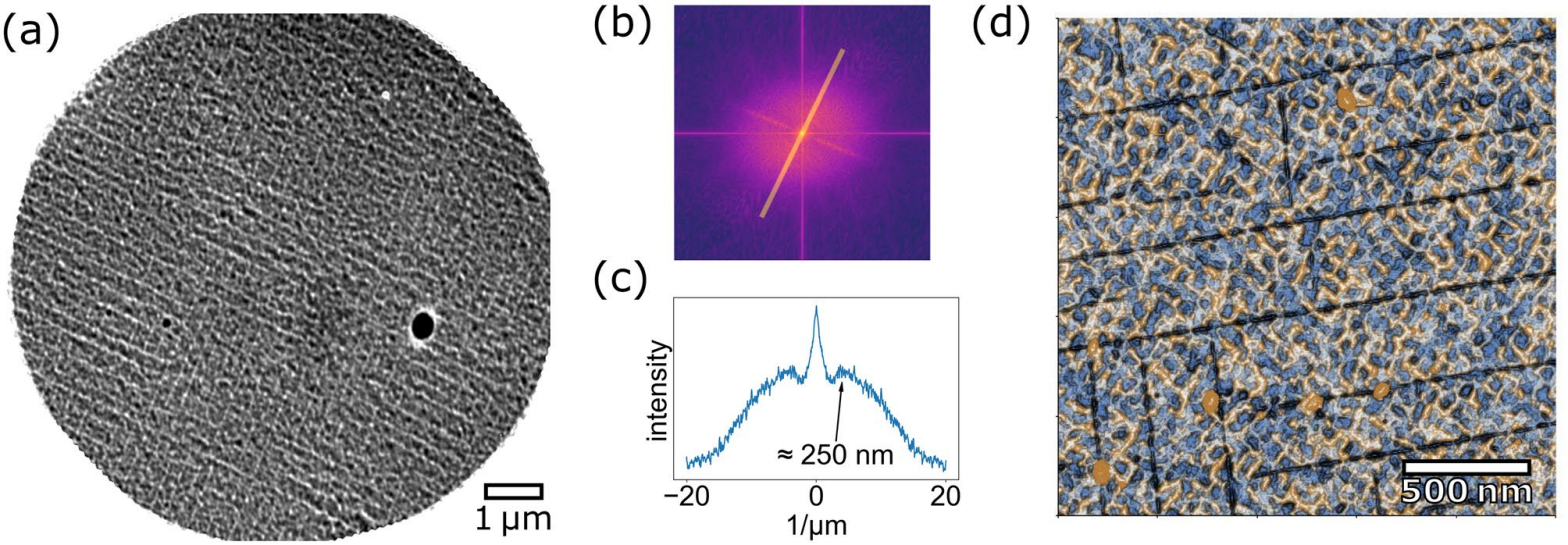
Cracks formed by strain release under thermal cycling. LEEM/MEM image recorded $1.5\,\text{eV}$ (**a**). The fast Fourier transform exhibits corresponding streaks of intensity (**b**), the maxima in the highlighted profile indicate a quasi-periodic spacing of $\approx 250\,\text{nm}$ (**c**). The AFM image (**d**) confirms these lines as topographic cracks and depicts the ripple texture of the stepped (001) surface state as substructure with smaller lateral dimensions.

### Strain-induced cracking

Beyond the faceting of the $\text{VO}_2(001)$ surface, the morphology features also stripe-like patterns that have been revealed by LEEM in the mirror electron microscopy mode for the entire surface of the thick $\text{VO}_2/\text{TiO}_2(001)$ film, see Fig. 4a. The emergence of stripe patterns has been reported for various $\text{VO}_{2}$ surfaces before: While (110) surfaces show a reversible formation of stripe patterns consisting of the competing $\text{VO}_{2}$ phases at the MIT^29–32^, particularly $\text{VO}_2/\text{TiO}_2(001)$ films can suffer permanent cracking along the ${<\!100\!>}$ directions, especially under the occurrence of large thermal stresses^18,31,33^. Similar to thin films, even $\text{VO}_{2}$ single crystals often crack when going through the MIT^17^.

In thin films, large thermal stresses can arise from the incompatible thermal contractions of the rutile $\text{TiO}_{2}$ substrate and the $\text{VO}_{2}$ film:^18^ The thermal expansion coefficient of rutile $\text{VO}_{2}$ is slightly higher than that of the substrate, ${\alpha _\text{VO}}_{2}^{\text{R}}>\alpha _{\text{TiO}_{2}}$. Therefore, beginning at the deposition temperature, tensile thermal stresses successively arise during cooling the sample. At the transition temperature, a drastic increase of the thermal expansion coefficient has been reported^34^, which is responsible for the observed cracking. In the monoclinic phase, however, $\alpha _{\text{VO}_{2}}^\text{M} <\alpha _{\text{TiO}_{2}}$ and thermal stresses are expected to decrease again under further cooling. Nagashima et al. proposed this mechanism of crack formation to occur when exceeding a critical thickness of $t_{cr} \approx 15\,\text{nm}$^18^. Interestingly, this characteristic thickness was found to be in very good agreement with values estimated according to fracture mechanics as $t_{cr} = 0.5 (k_f/\sigma )^2$, where $k_f$ denotes the fracture toughness and $\sigma$ the tensile stress^35^. These considerations further predict a minimal spacing $\lambda =8t(1-\sqrt{1-t_{cr}/t})$ between the cracks. Yet, random-like crack distributions have been observed with rather small densities, like $\approx 22.000/\text{mm}^{-2}$ for an exemplary film thickness of $30\,\text{nm}$, corresponding to crack spacings on a micron scale instead of the expected value $\lambda \approx 76\,\text{nm}$. Slightly higher crack densities of random distribution were shown by Paik et al^33^. Here, we report a significantly higher density and regular distributions for the MAD-grown film, caused by repeated slow cooling cycles down to a temperature of $77\,\text{K}$, for resistivity measurements. Note that the lower base temperature achieved in the present study, as explained above, should not lead to higher thermal stresses or, respectively, a smaller $t_\text{cr}$ than in the previous studies.

The cracks have been found to cover the entire sample surface, visible as an almost periodic, crosshatch-like texture in LEEM (Fig. 4a). Additional AFM measurements topographically verified the lines to be cracks (Fig. 4d). On smaller lateral scale, AFM depicts the rippled surface state described above with dominant features appearing rotated by $45^\circ$ with respect to the cracks, i.e. being aligned to the rutile $<\!110\!>$ directions. The faces of these cracks are oriented perpendicular to the film surface and hence invisible in LEED patterns, in contrast to the aforementioned facets identified above by $\upmu$LEED. As expected, the facet pattern appears to be interrupted a posteriori by the cracks as it has been formed already during deposition. Finally, it should be noted that samples, for which the formation of facets could be entirely avoided during growth, do show similar facet reflections in LEED after cracking^23^. Therefore, due to the large crack density, also facets located along the crack edges may have contributed to the RSMs shown above and could significantly enhanced their visibility in comparison to the uncracked rMBE samples.

In order to estimate the typical spacing in between adjacent cracks, a fast Fourier transform of the LEEM image has been evaluated (Fig. 4b). The profile along the highlighted path is shown in Fig. 4c, with maxima at $\pm 4\,\upmu \text{m}^{-1}$ corresponding to a crack spacing of $\approx 250\,\text{nm}$. Much closer to the theoretical minimal value of $67\,\text{nm}$ for the given film thickness, one now needs to consider a higher level of stress relaxation due to the formed cracks. As stated above, already for the initial sample state the metal-insulator transition occurs close to the bulk transition temperature of $340\,\text{K}$, indicating that some epitaxial stress was already released via interfacial defect formation during growth and annealing^36^. Nonetheless, as-grown samples did not show cracks in AFM (see Supplementary Fig. S3). Hence, considering only a reduced residual stress after annealing may account for the observed larger crack spacing with respect to the theoretical expectations. Although systematic investigations on the dependency of the crack densities on the sample dimensions, strain environments and process parameters are required to fully capture this phenomenon, the present results demonstrate that much higher densities can occur and lead to regular, quasi-periodic arrangements of cracks. Despite the regular appearance of the crack pattern, however, their formation ist still interpreted as an inherently random process: Cracks are assumed to nucleate first at such crystal defects that are located in highly strained sample regions. The propagation of crack lines is terminated at sample imperfections as well, most significantly at pre-existing cracks running in the respective perpendicular orientation. Consequently, the crack lengths are random, with rather long crack lines being created first. As the formation of a crack releases stress from its environment, additional, parallel crack defects will only nucleate beyond a characteristic minimal distance, which subsequently results in the quasi-periodic crack arrangement.

On the one hand, such cracking tendencies must be extensively explored, primarily as they hinder technological approaches to tune the MIT characteristics by epitaxial strain. On the other hand, many investigations on the bulk behavior of $\text{VO}_{2}$ may also benefit from this opportunity to prepare bulk-like specimen by rMBE means that do not fully disintegrate (like single crystals) but still provide a defined crystallographic orientation (unlike powder samples). Beyond that, this behavior may even be exploited in applications as a means for mesoscale self-organized patterning.

## Conclusions

We have presented a morphological characterization of $\text{VO}_{2}/\text{TiO}_{2}(001)$ films. As known for $\text{TiO}_{2}(001)$, a faceting tendency has been observed and the dominant facet types have been crystallographically identified. Further, it has been demonstrated that the ideal (001) surface can yet be obtained at moderate temperatures, showing large, atomically flat terraces. LEED investigations showed no fingerprints of the structural transition from rutile to monoclinic symmetry. Finally, a temperature induced cracking due to tensile epitaxial strain was found to form a quasi-periodic pattern of islands that show bulk-like transition properties.

## Methods

Two different types of epitaxial $\text{VO}_2/\text{TiO}_2(001)$ films were investigated: First, ultrathin films ($\approx 5\!-\!10\,\text{nm}$) have been deposited in an ultrahigh vacuum (UHV) system by reactive molecular beam epitaxy (rMBE) with an Epi UNI-Bulb RF plasma source for generating atomic oxygen. This growth recipe was largely inspired by studies using ozone as activated oxygen source^33,37^. Vanadium was evaporated by electron-beam heating and deposited onto the sample at a substrate temperature of $200\,^\circ \text{C}$ and under permanent atomic oxygen exposure. For this, the plasma source was operated at $350\,\text{W}$ and with a constant $\text{O}_2$ background pressure of ${5\cdot 10^{-5}\,\text{mbar}}$. As suggested in preceding growth studies^33^, deposition was followed by a post-anneal, here to $300\,^\circ \text{C}$ to enable a coalescence of the $\text{VO}_{2}$ grains. The stoichiometry of the samples has been verified by x-ray photoelectron spectroscopy (XPS), showing a separation of $14.3\,\text{eV}$ between the V1s and O2p peaks, characteristic for the $\text{V}^{4+}$ oxidation state^38^. No contamination was detected by XPS and a degradation of the surface by exposure to ambient atmosphere can be excluded. The surfaces of these samples have been morphologically characterized in situ by conventional low-energy electron diffraction (LEED) and scanning tunneling microscopy (VT-STM by Omicron).

The thicker film ($\approx 85 \,\text{nm}$) was prepared by metal-organic aerosol deposition^19^ (MAD). As precursor, vanadium acetylacetonate was solved in dimethylformamide, which was then sprayed onto the heated $\text{TiO}_2(001)$ substrate ($560\,^\circ \text{C}$) under near-ambient oxygen partial pressure. This sample was characterized ex situ by low-energy electron microscopy (LEEM) using an Elmitec LEEM III instrument and atomic force microscopy with an NX10 by Park Systems. Diffraction patterns of confined regions were recorded ($\upmu$LEED) from an illuminated sample area of $5\,\upmu \text{m}$ in the LEEM instrument. In comparison to the conventional LEED studies performed in situ on the rMBE samples, the $\upmu$LEED not only introduces spatial resolution but additionally provides a higher resolution in reciprocal space and a larger dynamic range. A comparison of these two sample types also allows for excluding effects specific to the different growth methods. All corresponding crystal structures shown in this article were drawn using the VESTA software^24^.

The ultrathin rMBE samples were prepared at rather low temperature ($200\,^\circ\text{C}$), whereas the thicker MAD film was deposited at a substrate temperature of $560\,^\circ \text{C}$. Due to the lower growth temperature, which ensures a minimization of interdiffusion between film and substrate, a grainy surface was observed by STM for the ultrathin films (see Supplementary Fig. S2) and no LEED pattern could be observed directly after growth. The rMBE sample preparation was hence finalized by a post-annealing step $300\,^\circ \text{C}$ after which clear LEED reflections are visible. As the MAD samples were deposited at a much higher temperature, no additional annealing was required in this case to improve the sample morphology. Nonetheless, only very diffuse diffraction spots were observed after sample transfer. This might be due to contaminations from ambient atmosphere and a sample surface which was overoxidized by air exposure. Therefore, also this sample was annealed at $300\,^\circ \text{C}$ and sharp diffraction patterns could be observed afterwards.

## Supplementary information

Supplementary material 1

Supplementary material 2
