# Supplementary material for: The morphology of VO2/TiO2(001): terraces, facets, and cracks
Source: Sci Rep. 2020 Dec 23;10:22374. doi: 10.1038/s41598-020-78584-9 (PMC7758337; doi:10.1038/s41598-020-78584-9)
Supplement: Supplementary file 1 — Supplementary material 1 [file 41598_2020_78584_MOESM1_ESM.pdf]

Supplementary Information

The morphology of $VO_2/TiO_2(001)$ — terraces, facets, and cracks

Jon-Olaf Krisponeit, Simon Fischer, Sven Esser, Vasily Moshnyaga, Thomas Schmidt, Louis F. J. Piper, Jan Ingo Flege, and Jens Falta

Contents

- Supplementary Figures 2-4
- Supplementary Video Legend 5

Supplementary Figures

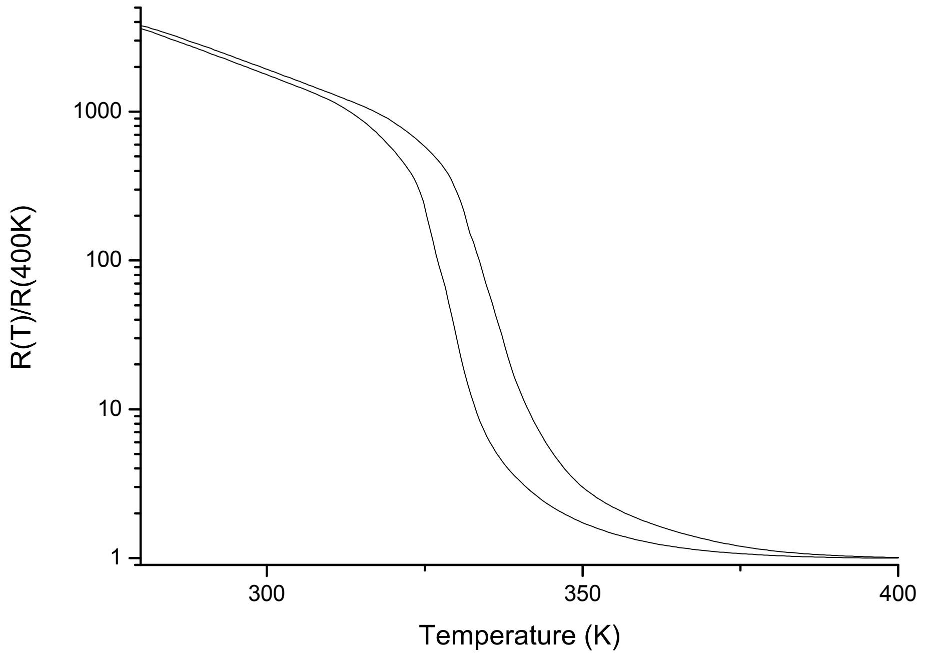

Supplementary Fig. S1: Temperature-dependent resistivity curve of a $VO_2/TiO_2(001)$ film grown by metal-organic aerosol deposition. The metal-insulator transition occurs close to the T_{MI} of bulk VO_2 (340 K) and hence indicates a release of epitaxial stress. Consequently, the rather broad hysteresis is attributed to a heterogeneous sample state originating from partial interfacial strain relaxation.

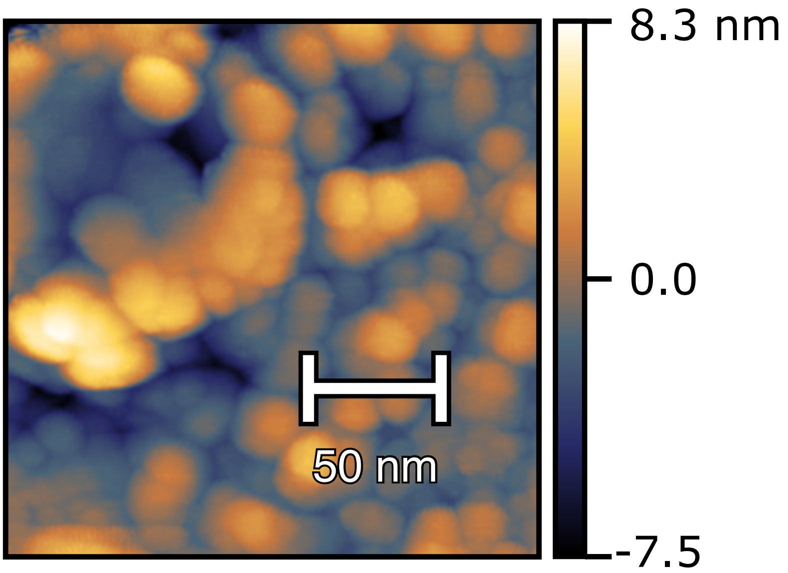

Supplementary Fig. S2: Scanning tunneling microscopy image of a VO_2 thin film deposited by reactive molecular beam epitaxy. Due to the corrugated morphology, a post-anneal is necessary in order to coalesce the grains and obtain a flat (001) termination.

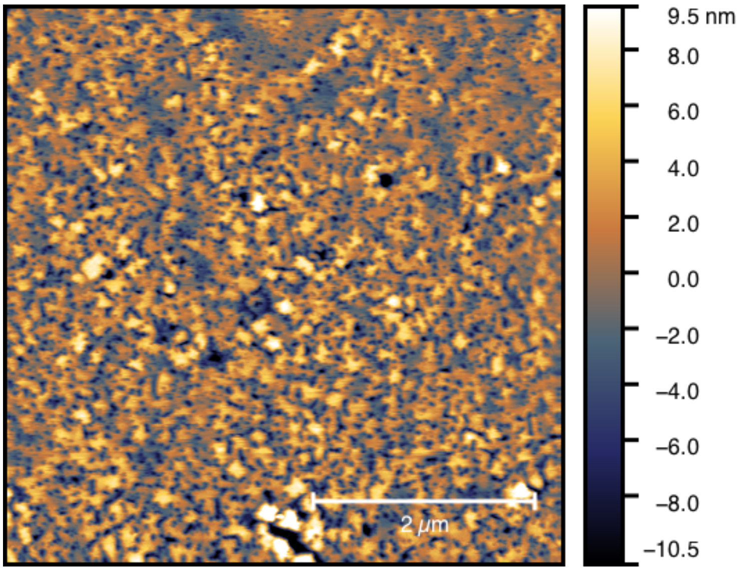

Supplementary Fig. S3: Atomic force microscopy image of an as-grown film prepared by metal-organic aerosol deposition. The morphology shows no cracks, contrasting the results for samples after additional thermal cycling (Fig. 4 in the report).

Supplementary Video Legend

Supplementary Video 1: A stack of µLEED images was recorded on the thick VO_2 film prepared by metal-organic aerosol deposition. Electron energy was increased from 6 eV to 25 eV in steps of 0.5 eV. With increasing energy the Ewald's sphere visible in the diffraction images is expanded. Reflections at fixed positions are relate to a (1x1) reconstruction of the rutile (001) termination. In addition, also facet reflections, which move with varying electron energy, are observed. Reciprocal space maps have been generated from this image stack and the dominant facet types have been identified (Fig. 3 in the report).
